# Categorization of allergic disorders in the new World Health Organization International Classification of Diseases

**DOI:** 10.1186/2045-7022-4-42

**Published:** 2014-11-28

**Authors:** Luciana Kase Tanno, Moises A Calderon, Bruce J Goldberg, Cezmi A Akdis, Nikolaos G Papadopoulos, Pascal Demoly

**Affiliations:** Hospital Sírio Libanês, São Paulo, Brazil; Section of Allergy and Clinical Immunology, Imperial College London, National Heart and Lung Institute, Royal Brompton Hospital, London, UK; Director, Kaiser-Permanente Southern California Regional Allergy-Immunology Laboratory, Consultant terminologist, International Health Terminology Standards Development Organization, Los Angeles, CA USA; Swiss Institute of Allergy and Asthma Research (SIAF), University of Zurich, Christine Kühne-Center for Allergy Research and Education, Davos, Switzerland; Department of Allergy, 2nd Pediatric Clinic, University of Athens, Athens, Greece; UPMC Paris 06, UMR-S 1136, IPLESP, Equipe EPAR, University Hospital of Montpellier, Montpellier, and Sorbonne Universités, 75013 Paris, France

**Keywords:** Allergic diseases, Classification, International Classification of Diseases (ICD), Hypersensitivity diseases

## Abstract

**Background:**

Although efforts to improve the classification of hypersensitivity/allergic diseases have been made, they have not been considered a top-level category in the International Classification of Diseases (ICD)-10 and still are not in the ICD-11 beta phase linearization. ICD-10 is the most used classification system by the allergy community worldwide but it is not considered as appropriate for clinical practice. The Systematized Nomenclature of Medicine Clinical Terms (SNOMED CT) on the other hand contains a tightly integrated classification of hypersensitivity/allergic disorders based on the EAACI/WAO nomenclature and the World Health Organization (WHO) may plan to align ICD-11 with SNOMED CT so that they share a common ontological basis.

**Methods:**

With the aim of actively supporting the ongoing ICD-11 revision and the optimal practice of Allergology, we performed a careful comparison of ICD-10 and 11 beta phase linearization codes to identify gaps, areas of regression in allergy coding and possibly reach solutions, in collaboration with committees in charge of the ICD-11 revision.

**Results:**

We have found a significant degree of misclassification of terms in the allergy-related hierarchies. This stems not only from unclear definitions of these conditions but also the use of common names that falsely imply allergy. The lack of understanding of the immune mechanisms underlying some of the conditions contributes to the difficulty in classification.

**Conclusions:**

More than providing data to support specific changes into the ongoing linearization, these results highlight the need for either a new chapter entitled Hypersensitivity/Allergic Disorders as in SNOMED CT or a high level structure in the Immunology chapter in order to make classification more appropriate and usable.

## Current status of the international classification of diseases (icd) for allergic disorders

### Background

#### The need for a classification of hypersensitivity/allergic diseases

Allergic diseases can be expressed in many different organs and in any age group, having a significant impact on the quality of life of patients and their families. Every health professional can face both mild and severe allergic conditions and it is believed that the world is dealing with a real allergy epidemic, resulting in considerable consequences for patients around the world and in important costs for societies [1]. The classification of hypersensitivity/allergic diseases, supported by updated knowledge of the underlying mechanisms, helps to understand the ongoing scenario and to potentially assist in the development of new diagnostic and management approaches for these conditions.

**Figure 1:**
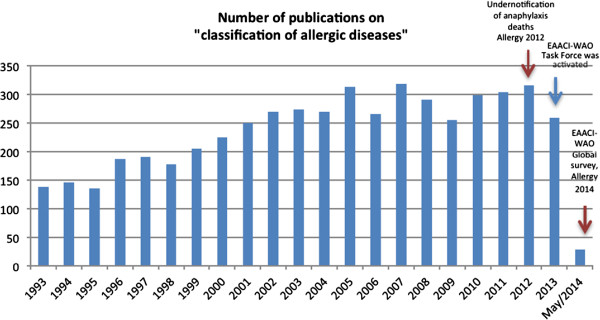
**Number of publications on “classification of allergic diseases” per year (1993-2014), according to a general search in the PUBMED website.**

During recent years, the allergy community has made efforts to work on the classification of specific clinical hypersensitivity/allergic diseases. These disorders represent what is commonly encountered in daily clinical practice and are included in regular continuous medical educational programs in allergy meetings and congresses. When searching for “classification of allergic diseases” in PUBMED, the number of publications has tripled during the last decade, regardless to the type/quality of the papers (Figure 1). However, currently there is no universal standard platform in which allergic diseases are classified to allow proper analysis of their impact in the medical community and the general population, allowing comparability accepted worldwide.

Considering that without a common understanding and a strict use of terms to define hypersensitivity/allergic diseases, neither science nor patient care could be optimal, the European Academy of Allergy and Clinical Immunology (EAACI) and the World Allergy Organization (WAO) have joined forces to propose a revision of the nomenclature for allergy for global use a few years ago [2] but did not attempt at that time to liaise with the World Health Organization (WHO) International Coding of Diseases (ICD).

#### The WHO International Classification of Diseases

The International Classification of Diseases (ICD) is the world’s standard tool to capture mortality and morbidity data maintained by the World Health Organization (WHO). This information is used to organize and code health data that is used for statistics and epidemiology, health care management, allocation of resources, monitoring and evaluation, research, primary care, prevention and treatment. It attempts to provide a picture of the general health situation of countries and populations as well as standardize a common language for reporting and monitoring diseases. The ICD is revised periodically and the 11^th^ revision is ongoing.

The ICD is the most used classification system by the allergy community worldwide as demonstrated by the recent EAACI–WAO survey on the global classification and coding of hypersensitivity/allergic diseases [3], but it was not considered appropriate for clinical practice by the majority of participants. The majority of the responders in this survey considered it neither easy nor accurate to classify hypersensitivity/allergic diseases.

We have demonstrated that ICD-10 does not allow access to reliable mortality data about some important allergic conditions such as anaphylaxis [4]. If hypersensitivity/allergic diseases are not adequately coded in ICD, this will result in misclassification, leading to low visibility of these conditions and therefore, inaccurate data capture.

#### The systematized nomenclature of medicine–clinical terms

The Systematized Nomenclature of Medicine Clinical Terms (SNOMED CT) is an international reference terminology with an ontological underpinning used for decision support, outcomes analysis and interoperability. It is derived from the merger of SNOMED RT (Reference Terminology), a product of the college of American pathologists and the Read codes, a product of the national health service of the United Kingdom. In April 2007, SNOMED-CT intellectual property rights were transferred to the not-for-profit association International Health Terminology Standards Development Organization (IHTSDO, http://www.ihtsdo.org/).

Allergy terminology in SNOMED is based on the EAACI-WAO nomenclature and is organized as an upper level hierarchy under disease called hypersensitivity condition, thus enabling the representation of hypersensitivity propensities, processes (reactions) and diseases for use in electronic health records [5].

Although SNOMED CT is still not globally used and the ICD-11 linearization structure remains under construction, the WHO – IHTSDO joint advisory group plans to align ICD-11 with SNOMED CT so that they share a common ontological basis [6]. For this reason, it would be advantageous to take the example of the representation of hypersensitivity diseases in SNOMED CT to improve allergological representation in the ICD-11.

#### The ICD-10 and the ICD-11 revision

Work on ICD-10 began in 1983 and was fully developed by 1992 in order to track health statistics. ICD-10 is the 10^th^ revision of the International Statistical Classification of Diseases and Related Health Problems. It accounts for more than 17,000 codes used by 25 countries for reimbursement and resource allocation in the health system and by 110 countries for reporting cause of death reporting and statistics. The hypersensitivity/allergic diseases are not considered as a separate category in ICD-10.

The 11^th^ ICD was officially launched by WHO in March 2007 and the final draft is expected to be submitted to WHO’s World Heath Assembly for official endorsement by 2017. Different from the ICD-10, which was presented as just title headings, the proposed structure of ICD-11 includes definitions for each entity and follows a content model.

We were able to highlight the “misclassification” of allergic diseases in ICD-10 by the results of our community survey [3]; however, when the beta phase of ICD-11 discussion was made available online in May 2012, the linearization was pre-established by inheriting some ICD-10 characters and developing some innovative aspects. A proper classification/coding of hypersensitivity/allergic diseases by clinical entity, by etiology or by symptom is still missing in ICD-11 and incompletely classified by the ICD-11 Topic Advisory Groups (TAGs) as of May 2014 [7].

Taking the above into account and with the aim of active support of ICD-11 changes in favor of patients with hypersensitivity/allergic diseases and improved practice of Allergology and Clinical Immunology, we present a careful comparison of ICD-10 (2010 version) and the ICD-11 beta phase linearization codes (May 2014 version) in order to identify any gaps and areas of regression in allergy coding and possibly achieve solution with members of the ICD-11 TAGs.

## Methods

### Comparison between ICD-10 and ICD-11 beta phase linearization for better coding of allergic diseases

#### The process of comparison

In the first phase of the process, we were able to prepare a list of the important hypersensitivity/allergic diseases (key words) that health care professionals are dealing with on a daily basis. The EAACI-WAO revised nomenclature [2] was the basis of the proposed categories, updated by new knowledge generated since. It was, then validated by a core group (LKT, MAC, PD) and cross-checked to avoid overlapping terms and words able to provide dubious interpretation of data. ***“Hypersensitivity”*** is defined as “conditions clinically resembling allergy that cause objectively reproducible symptoms or signs, initiated by exposure to a defined stimulus at a dose tolerated by normal subjects” and “***allergy”*** as “a hypersensitivity reaction initiated by immunologic mechanisms” [2]. However, these concepts can generate some misunderstanding between non-specialists, consequently the authors decided to always link the terms hypersensitivity and allergy across the current paper. For the purpose of the current paper this simplification is used, however, the authors are aware that hypersensitivity is not identical to allergy. In addition, notwithstanding the importance of immunological diseases such as primary and secondary immunodeficiencies and autoimmune diseases, we did not include these conditions herein, as they do not pose similar coding problems.

In the second phase of this project, to better construct and appraise the proposed comparison between the ICD-10 and the 11 beta phase on hypersensitivity/allergic diseases, all possible and relevant corresponding codes and classifications from the ICD-10 [8] and the ICD-11 beta phase linearization [7] were selected. All the process of searching the key words correspondences from both ICD-10 and 11-beta draft (May 2014 version) had an online basis. With the aim of increasing the accuracy of the search, an independent reviewer crosschecked all codes. We are however aware that the definitive codification is not established in the linearization up to now.

The preparation of the whole scheme containing all the list of hypersensitivity/allergic diseases and corresponding ICD-10 and 11 codes provided us a picture for further evaluation. The document was extensively analyzed to go through the topics and have a real understanding of the innovations, map the gaps and trade-offs and find the missing and/or imprecise terms of the ICD-11 beta phase linearization as well as provide guidance for the construction of a realistic global, cross-cultural, multi-axial classification system useful for allergists, non-allergists and non-physicians. Based on this analysis, we were able to look for feasible solutions and suggest changes to improve the positioning of allergic diseases into the ongoing ICD-11 discussion.

## Results and discussion

### Lessons of the ICD comparison for hypersensitivity/allergic diseases

#### Main hypersensitivity/allergic diseases categories

The outcome of the above process was the generation of a list of 192 key words related to 10 main categories of hypersensitivity/allergic diseases covering respiratory (asthma and rhinitis), skin (dermatitis, urticaria, angioedema), ocular (conjunctivitis) and complex and/or multi-system diseases (drug, food and hymenoptera allergies and anaphylaxis). Based on the analysis of these words into the ICD-10 and 11 beta phase, we could identify the main gaps and trade-offs.

#### Gaps related to hypersensitivity/allergic diseases in ICD-10 and ICD-11 beta

We noticed improvements in ICD-11 beta with regards to hypersensitivity/allergic diseases; indeed, it was evident that several clinical patterns are scattered in many different chapters across ICD-10 such as food and drug hypersensitivity (Table 1). However, more than 3 different clinical presentations (*e.g.,* “anaphylactic shock non-specified” and “angioneurotic oedema”) are still inappropriately classified under the same sub-chapter of ICD-11 beta (T78 of Chapter XIX Injury, poisoning and certain other consequences of external causes), which is also not considered by WHO rules as a valid underlying cause of death, although it clearly is [4].

**Table 1:** **Gaps and/or misclassification of the main hypersensitivity/allergic disorders described on the EAACI–WAO revised nomenclature of allergy scattered across the ICD-10 chapters and sub-chapters (version 2010)**

Hypersensitivity disorders (according to the*EAACI-WAO revised nomenclature + updates)*		ICD-10 version 2010 corresponding chapter(s)	Main related sub-chapter(s) according to the ICD-10
Asthma		Chapter X: Diseases of the respiratory system	J45.0 - J45.9, J46
Rhinitis		Chapter X: Diseases of the respiratory system	J30.0 – J30.4, J31, J33
Conjunctivitis		Chapter VII Diseases of the eye and adnexa	H10.0 – H10.9, H16.2 – H16.9, H18.6
Skin diseases	Dermatitis	Chapter XII Diseases of the skin and subcutaneous tissue	L20 – L30
	Urticaria	Chapter XII Diseases of the skin and subcutaneous tissue	L50.0 – L50.9
	Angioedema	Chapter XIX Injury, poisoning and certain other consequences of external causes	T78.3
		Chapter III Diseases of the blood and blood-forming organs and certain disorders involving the immune mechanism	D84.1
Food hypersensitivity		Chapter XIX Injury, poisoning and certain other consequences of external causes	T78.0, T78.1
		Chapter XII Diseases of the skin and subcutaneous tissue	L23.6
		Chapter XI Diseases of the digestive system	K52.2, K52.3, K52.8, K52.9
		Chapter XXI Factors influencing health status and contact with health services	Z71.3
Drug hypersensitivity		Chapter XIX Injury, poisoning and certain other consequences of external causes	T88.1, T88.2, T88.6-T88.8
		Chapter XX External causes of morbidity and mortality	Y40.0 – Y59.9
		Chapter XII Diseases of the skin and subcutaneous tissue	L51 – L52
		Chapter XXI Factors influencing health status and contact with health services	Z88.0 – Z88.9
Venom hypersensitivity		Chapter XX External causes of morbidity and mortality	X23, X25, T63
Anaphylaxis		Chapter XIX Injury, poisoning and certain other consequences of external causes	T78.0 – T78.2

**Figure 2:**
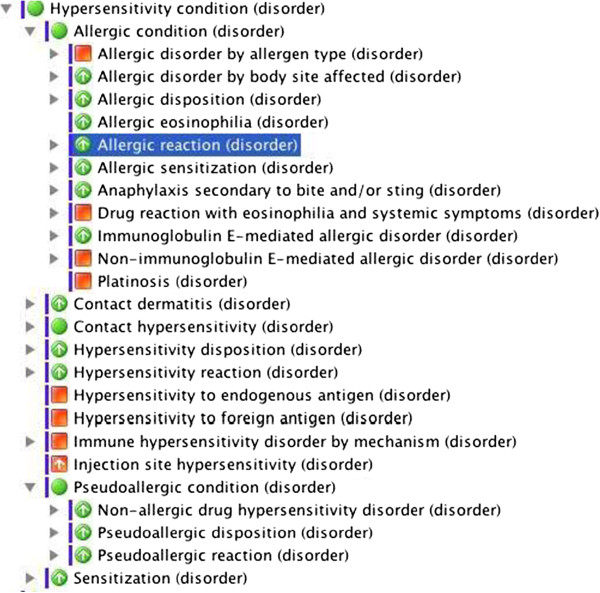
**Upper level hierarchies representing hypersensitivity and allergic disorders in SNOMED CT.**

In terms of respiratory diseases, both asthma and rhinitis were categorized in ICD-10 and ICD-11 beta basically by time patterns (such as intermittent and persistent) and severity (only for asthma) with no reference to the underlying mechanisms. We also noticed the absence of important etiologies as the content model of these classifications concentrated in vague causative descriptions (“Allergic rhinitis due to pollen”, “Other seasonal allergic rhinitis” and “Other allergic rhinitis”), different from what is properly listed in SNOMED CT (Figure 2). In the May 2014 version of the ICD-11 beta draft, some aspects such as exercise-exacerbated conditions, overlapping conditions (*e.g.,* Asthma induced by Aspirin and Exercise-induced anaphylaxis dependent on food) and an updated classification for occupational rhinitis and asthma are still absent. Meanwhile, for ocular allergy, the ICD-10 and ICD-11 beta codes/classification have focused in substance by site (*e.g.,* Disorders of the eyeball – anterior segment); references to cause and underlining mechanisms are still missing.

We observed improvements in the classification of skin allergies in ICD-11 beta compared to ICD-10 (*e.g.,* inclusion of a more detailed contact dermatitis and atopic eczema classification), but some systemic conditions such as drug-induced anaphylaxis are still scattered into the chapter “Disorders of the Skin”. Although many drug allergies may have a cutaneous clinical presentation, the drug hypersensitivity/allergic disorders can be considered one of the most complex conditions once the many factors involved in the pathogenesis are considered [9], suggesting the need for a “Complex Hypersensitivity/Allergic Conditions” grouping. The “Diseases of the Skin” chapter also incorporated the classification of insect bite, venom or bite allergic reactions in the ICD-11 linearization. We noticed that the new classification proposed was more detailed, trying to associate the clinical presentation and the cause such as for allergic contact dermatitis; however, systemic reactions were once again lacking. In ICD-10, these allergic conditions were improperly classified under the Chapter XX External causes of morbidity and mortality, sub-chapter “Contact with venomous animals and plants”. The term “contact” can allow multiple interpretations that can vary from a direct cutaneous contact, such as an IgE-mediated contact urticaria or a type IV contact dermatitis.

**Figure 3:**
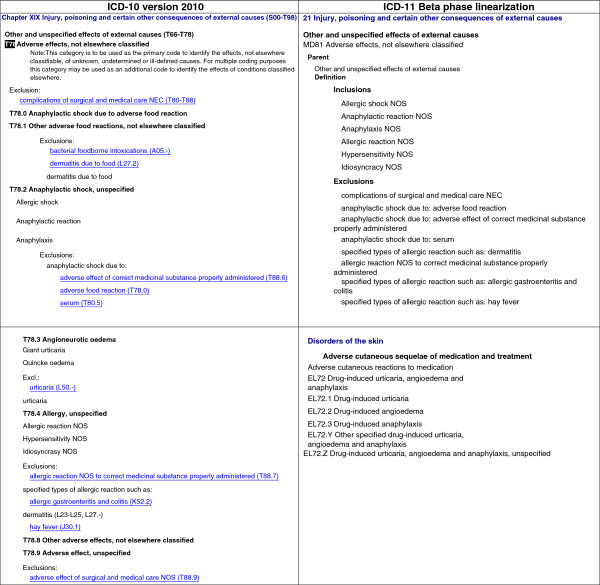
**Examples of the inadequacy of the classification of anaphylaxis, drug and food allergy in ICD-10 and ICD-11 Beta phase linearization.**

For anaphylaxis, the situation is not improved in ICD-11 beta compared to ICD-10; indeed, in both classifications, anaphylaxis still resides under the same nonspecific “Chapter XIX Injury, poisoning and certain other consequences of external causes” which the main references are “Anaphylactic shock due to adverse food reaction” (T78.0) and “Anaphylactic shock, unspecified” (T78.2). Anaphylaxis is defined as a “severe, life-threatening systemic hypersensitivity reaction” [10], but we could identify some specific anaphylaxis conditions under the Skin Disorders chapter, such as “anaphylaxis due to contrast media” listed into the “Miscellaneous of urticarial disorders” (Figure 3). Moreover, the term “shock”, which means the drop of blood pressure, is one of the anaphylaxis manifestations, however, should not be taken as synonymous or always together. It suggests the need of implementing a severity degree classification for anaphylaxis into the ICD.

#### Trade-offs identified by the ICD comparison for hypersensitivity/allergic diseases

Knowledge generated since the EAACI-WAO revised nomenclature publication [2] can be used to update outdated concepts and terms still present in the ICD-11 beta phase. An example of this is the term “vasomotor allergic rhinitis” [11–13]. In the same way, the use of some synonyms and narrow terms in the ongoing ICD-11 beta revision may need update, such as the concepts of intrinsic and extrinsic asthma, which has not been favored by the relevant medical communities, following the understanding of asthma as a multi-phenotype disease [13–17]. Published classifications in specific areas of the allergy field can support these changes [9–36]. For that, the many allergy position papers and international consensus should be considered [9–36].

Another trade-off in ICD-10 and ICD-11 is in the area of drug allergy in which the mechanisms, clinical presentations and causes are diluted within the ICD structure. Clinical presentations remain scattered among many chapters such as “Diseases of the Skin” and “External Causes”; whereas drug allergy causes are still listed in the “External Causes” chapter, under the “Complication of Medical and Surgical Care” category and not linked to the clinical presentation or related mechanism. The preceding may indicate the need for many different codes to better characterize a reaction. This is a real weakness of the ICD, preventing good statistics of one of the most common drug side effects.

## Conclusions

### Outcomes of the ICD comparison for hypersensitivity/allergic diseases

There is a significant degree of misclassification of terms in the hypersensitivity/allergic - related hierarchies stemming not only from unclear definitions of these conditions but also from the use of common names that falsely imply allergy, as well as a lack of understanding of the immune mechanisms underlying some of the concepts (Figure 3). Many of these issues have been corrected in SNOMED CT, but an appropriate hypersensitivity/allergic diseases classification is still missing in the (May 2014 version) ICD-11 structure.

We have highlighted the need for updating the hypersensitivity and allergy conditions classification in the ICD-11 beta revision by objectively examining evidences of misclassification and missing and/or imprecise terms, which may hamper the reliable documentation and analysis of allergy data worldwide. This scenario is particularly notable for some complex conditions, such as drug allergy, food allergy and anaphylaxis requiring the revision of the current structure of the ICD.

We believe that more than providing data to support specific changes into the ongoing linearization, these results highlight the need for either a new chapter in ICD-11 possibly entitled Hypersensitivity/Allergic Disorders or at the very least the aggregation of all such diseases under the “Diseases of Immune System” chapter in order for the overlaps to be double parented to the appropriate ‘system’ chapters. Due to the previously mentioned major problems, post-coordination with the existing codes will not be sufficient. In this way, based on the discussions with some active representatives of the groups in charge of the ICD-11 beta revision (Topic Advisory Groups) to have achievements to the Hypersensitivity/Allergic Diseases classification into the ICD-11, they are aware that ICD-10 deals poorly with allergy which does not fit neatly into a single body system and has not been properly represented within the revision process. In addition, representation by allergy specialists in the ICD-11 revision process is still a lacking.

In conclusion, the data presented here aim to help further discussion regarding the representation of allergy-related content in ICD-11 in order to improve the documentation and coding of this important domain of human health. This document will help us to bring up the discussion of the visibility of our specialty expecting for changes and to support the recognition of allergy and clinical immunology as a specialty by WHO. In addition, it may serve as the basis of a classification proposal ready for crowdsourcing the allergy community and to be shared with WHO committees for future endorsement.

## Abbreviations

EAACI: European academy of allergy and clinical immunology
ICD: International classification of diseases
IHTSDO: International health terminology standards development organization
SNOMED CT: Systematized nomenclature of medicine clinical terms
SNOMED RT: Systematized nomenclature of medicine reference terminology
TAGs: Topic advisory groups
WAO: World allergy organization
WHO: World health organization.
